# Long persistence of EV71 specific nucleotides in respiratory and feces samples of the patients with Hand-Foot-Mouth Disease after recovery

**DOI:** 10.1186/1471-2334-10-178

**Published:** 2010-06-18

**Authors:** Jun Han, Xue-Jun Ma, Jun-Feng Wan, Ying-Hui Liu, Yan-Ling Han, Cao Chen, Chan Tian, Chen Gao, Miao Wang, Xiao-Ping Dong

**Affiliations:** 1 State Key Laboratory for Infectious Disease Prevention and Control, National Institute for Viral Disease Control and Prevention, Chinese Center for Disease Control and Prevention, Beijing, China; 2 Fuyang Municipal Center for Disease Control and Prevention, Fuyang, Anhui, China

## Abstract

**Background:**

EV71 is associated with the fatal cases of brain stem encephalitis during large HFMD outbreaks from 1998 to 2008. EV71 may continuously shed from upper respiratory tracts and feces of HFMD patients for relatively long time after recovery. However, the persistence of viruses in the patients' secretions and excretions is not clear.

**Methods:**

Serial throat swabs and feces of 34 definitely diagnosed patients, including 30 mild cases and 4 severe cases, were traced and collected with the interval of 2 to 4 days for up to 32 and 48 days, respectively, and tested by a nested RT-PCR.

**Results:**

The EV-71 specific sequences were identified by a Nested RT-PCR in all specimens of 0-4 days, and 5-8 days. The positive rates of EV71 in throat swabs dropped markedly to 42.86% during 9-12 days, and maintained at 20-30% during 13-24 days, while that in feces reduced to 71.43% during 9-12 days, and maintained roughly 20% till 37-40 days. EV71 nucleotide of 36.36% cases disappeared simultaneously both in throats and feces, 39.39% cases showed longer persistence of EV71 nucleotides in feces, and 21.21% were longer in throats. The longest duration of shedding observed was 24 days for throat swabs and 42 days for fecal specimens.

**Conclusions:**

EV71 shedding from respiratory tract may continue for nearly four weeks after onset, but its excretion through feces can persist more than five weeks.

## Background

Enterovirus 71 (EV71), which was firstly described in 1969 during an outbreak with central nervous system complications in California, causes a variety of neurological diseases, including aseptic meningitis, encephalitis, and poliomyelitis-like paralysis [1]. This virus is also one of only a few enterovirus serotypes most often associated with large outbreaks of hand, foot, and mouth disease (HFMD) [2,3]. EV71 is associated with the fatal cases of brain stem encephalitis during large HFMD outbreaks in Malaysia (1997) [4], Taiwan (1998, 2000 and 2001) [5-7], Australia (1999) [8] and Singapore (2000) [8,9]. In March 2008, an EV71 HFMD outbreak appeared in Fuyang City, Anhui Province of China, and subsequently spread quickly in almost all provinces in PR China [10].

Continuous circulation of EV71 in the community was evident, since EV71 has been repeatedly detected and isolated from HFMD outbreaks worldwide every year from 1998 to 2008 [7,9-11]. The transmission pathways of EV71 are believed to be multiple, i, e, close contact, droplets through respiratory tract and contaminated water through gastrointestinal tract [12]. HFMD patients and asymptomatic persons are the major infectious sources. Some studies have described that EV71 may continuously shed from upper respiratory tracts and feces of HFMD patients for relatively long time after recovery [11,13]. However, the persistence of viruses in the patients' secretions and excretions is not well-documented. In this study, serial throat and feces samples of 34 definitely diagnosed HFMD cases were collected during and after hospitalizations, including 30 mild cases and 4 severe cases. We found that EV71-related sequences persisted in the HFMD children for weeks after recovery, although about half of the cases showed EV71 negative conversion in the first two weeks after onset.

## Methods

### Sample collection

34 definitely diagnosed HFMD cases were enrolled in this study, who had typical clinical manifestations and hospitalized. Among them, 30 cases were mild and 4 were severe. The mean durations in hospital were 5.4 ± 1.2 days in mild group and 5.5 ± 2.4 days in severe one. All cases were EV71 RNA positive both in respiratory and feces samples when hospitalized. Medical follow-up and sampling for each patient was planned to last for 6 weeks after onset of the disease. Serial throat swabs and feces of each patient were collected every four days after onset. All specimens were immediately immersed into sterile tubes containing viral transport medium. The sample collection started from the beginning of May 2008 and the last case in this study was enrolled at the end of May. Total duration of sampling took roughly three months (from May to July, 2008).

### Automated RNA extraction

Prior to RNA extraction, feces samples were treated with chloroform according to the protocol described elsewhere [14]. Automated RNA extractions of all collected specimens were performed with MagneSil Total RNA mini-isolation kit (Promega), using the Biomek FX liquid handler (Beckman Coulter) according to the manufacturer's instructions. Briefly, 120 μl throat swabs or pretreated feces samples was mixed with 120 μl of lysis buffer containing carrier RNA and incubated at room temperature for 10 min. The lysed sample was then transferred to the 96 wells of a plastic plate with 20 μl of magnetic beads, followed by the process of automatic magnetic separation. RNA was recovered in 40 μl of elution buffer. The time elapsed from automated RNA extraction to PCR setting up was less than 35 min. Quality control did not show any trail of nucleic acids contamination among the samples after extraction by automated instruments.

### Nested RT-PCR for EV71

For amplification of EV71 specific fragment, a Nested RT-PCR was designed between VP3 to VP1 region of EV71 with the inner-primer-F (5'-CCATCCAGGGAGATAGGGTAG-3', nt. 2419-2440) and inner-primer-B (5'-CTGGAACCTTACCTGTGTCCA-3', nt. 2552-2573), as well as outer-primer-F (5'-GCAGCCCAAAAGAACTTCAC-3', nt. 2372-2392) and outer-primer-B (5'-ATTTCAGCAGCTTGGAGTGC-3', nt. 2578-2598). 11 μl of extracted RNA samples were subjected into the Nested RT-PCR as described below. Briefly, the reactions were carried out with One-Step RT-PCR kit (QIAGEN) on 7500 fast PCR instrument (Applied Biosystems), with an initial reverse transcription step at 42°C for 45 min, followed by PCR activation at 95°C for 10 min and 35 cycles of amplification (94°C for 30 s, 45°C for 30 s, 72°C for 30 s). Nested PCR assays were subsequently performed with 1:10 dilution of previous RT-PCR products on 7500 fast PCR instrument (Applied Biosystems), programmed with the following parameters (32 cycles of 94°C for 30 s, 45°C for 30 s and 72°C for 30 s). PCR products were observed in 2% agarose electrophoresis.

### Statistical analysis

The differences of the persistence of EV71 nucleotides between mild and severe cases, between genders and among the onset ages were performed with Chi-Square tests.

### Ethical approval

This study was approved by the Ethical Committee of National Institute for Viral Disease Control and Prevention, China CDC. Sample collection was agreed by child's parents or grandparents with informed consent.

## Results

A nested RT-PCR protocol for EV71 sequences had been set up, producing a 226 bp and a 154 bp product in the first and second PCR reaction, respectively. The sensitivity of this method for EV71 had been previously measured, which was able to detect 10^2 ^and 10^1 ^copies of virus based on PFU test. The specificity of this assay was tested with the RNAs extracted from other enteroviruses, including CA16, CA24, CB2-5, ECV9 and ECV30. No cross-amplification was obtained.

A total of 34 definitely diagnosed HFMD cases were subjected into this study. All cases had serial feces samples and 33 cases had serial throat swabs. The average ages were 2.3 ± 1.6 years old and gender ratio was 22:12. Mostly the first samples of the cases (31/34) were collected during the first week after onset based on the time of hospitalization. RT-PCR tests revealed that the first respiratory and feces samples of all cases were EV71 positive. The throat swabs and feces of these patients were traced and collected with the interval of 2 to 4 days and grouped each four days after onset. Each case contained at least five serial samples after the first one. The longest time of the collected throat swabs and feces were 32 and 48 days after onset, respectively.

**Figure 1:**
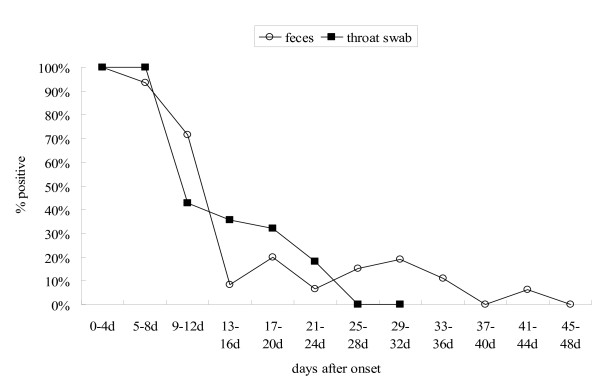
**The positive rates of EV71 nucleotides in samples from the definitely diagnosed HFMD patients**. The positive rates of EV71 related nucleosides identified in serially collected throats and feces samples from the definitely diagnosed HFMD patients. Square hole represents RT-PCR positive rates from feces sample. Black square represents RT-PCR positive rates from throat swabs.

Based on the grouping, each group had about twenty clinical samples. RT-PCR (Figure 1) showed that the EV71 specific sequences were identified in all specimens of 0-4 days, and 5-8 days after onset. The positive rates of EV71 in throat samples dropped markedly to 42.86% during 9-12 days, and maintained at about 20-30% during 13 to 24 days after onset, while that in feces reduced to 71.43% during 9-12 days, and maintained roughly 20% afterwards till 37-40 days after onset. EV71 associated nucleotides were undetectable in respiratory samples fourth weeks after onset and almost undetectable in feces more than five weeks later. The longest EV71-postive time in throat swab was 24 days and that in feces was 42 days post-onset.

**Figure 2:**
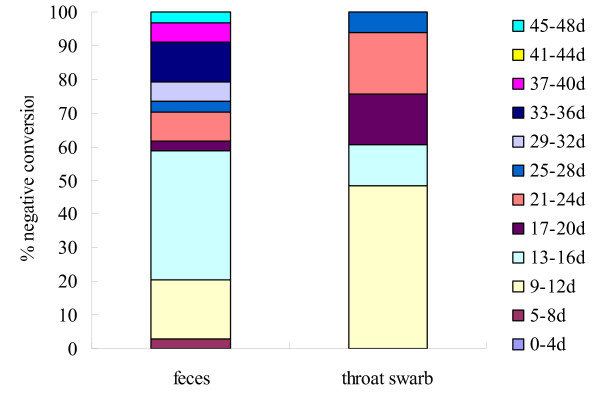
**The portions of negative conversion of EV71 nucleosides in the HFMD convalescent**. The portions of negative conversion of EV71 related nucleosides in throats and feces samples from the HFMD patients in the convalescent.

To evaluate the negative conversion rate in clinical samples of EV71 nucleotides after disease, the time period of EV71 negative closest to that of EV71 positive was taken as the time of EV71 nucleotide negative conversion. Figure 2 showed that none of the tested cases converted to EV71 negative in throats or in feces in the first four days. In the time period of 5-8 days, only one feces showed EV71 negative conversion. EV71 negative conversion happened mostly in the time periods of 9-12 days and 13-16 days after onset. 48.48% throat swabs and 17.65% feces samples in the period of 9-12 days, as well as 12.12% throat swabs and 38.24% feces samples in the period of 13-16 days were EV71 negative, respectively. Afterwards, the EV71 negative conversion rates increased gradually in throats and feces, but EV71 nucleotides in feces samples lasted clearly longer than that in throats. No significant difference in persistence of EV71 nucleotides were analyzed between mild and severe cases, between genders and among the onset ages.

**Figure 3:**
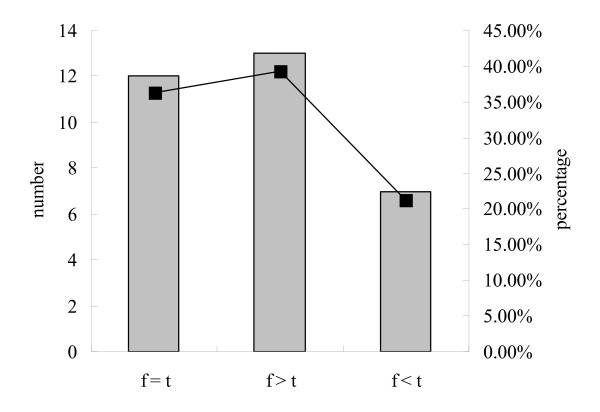
**Comparison of the clearances of EV71 in the throat and feces specimen of individual cases**. F: feces; T: throat. The right Y-axis represents the numbers of the cases and the left Y-axis represent the percentages.

To figure out the possible different profiles of EV71 disappearance, persistent states of EV71 nucleotides in throats compared to feces of individual patient were analyzed. 36.36% (12/33) cases revealed almost same time period of EV71 nucleotide disappearance both in throats and feces, 39.39% (13/33) cases showed longer persistence of EV71 nucleotides in feces, and 21.21% (7/33) were longer in throats (Figure 3). Furthermore, the identifications of EV71 nucleotides in each time period of individual case were counted. Only two out thirty-three cases showed EV71 positive in the subsequent samples after previous negative in their serial throat samples, however, 6 cases were EV71 positive reversion in the serial feces samples.

## Discussion

HFMD has been an important public health concern worldwide, especially in the Asia-Pacific region. Up to now, more than 900,000 HFMD cases have been reported in mainland of China. Enteroviruses can be isolated from both the lower and upper alimentary tract and can be transmitted by both fecal-oral and respiratory routes. Fecal-oral transmission may predominate in areas with poor sanitary conditions, whereas respiratory transmission may be important in more developed areas. The relative importance of the different modes of transmission probably varies with the particular EV and environmental setting [1]. Like other infections of enteroviruses, e.g. Coxsackie's viruses and polioviruses, EV71-caused HFMD often occurred sporadically or epidemically, sometimes without clear transmission chain [2,15]. One of the reasons is believed to be the wide and continuous circulation of EV71 among humans as well as environment [16,17]. On the other hand, long time of virus persistence and shedding in the patients' secretions and excretions makes infectious sources be more abundant. Additionally, relatively stronger resistance of viruses in environment let the disease more easily transmit [18].

With the established nested RT-PCR, we prove a long persistence of EV71 nucleotides in the throats and feces of the patients with HFMD. Only about half of the patients show EV71 negative in their respiratory secretions and stools two weeks after onset. The traditional techniques for detecting and characterizing enteroviruses rely on the time-consuming and labor-intensive procedures of viral isolation in cell culture and neutralization by reference antisera [1,19]. Virus persistent time in the throat in this study seems to be longer than that of previous data, mostly obtained from virus culture, which showed EV71 shedding up to 2 weeks [13]. Isolation of enteroviruses from specimens need appropriate cultured cell lines and suitable specimen. The best specimens for isolation of virus are, in order of preference, stool specimens or rectal swabs, throat swabs or washings, and cerebrospinal fluid. Throat swabs or washings and CSF are most likely to yield virus isolates if they are obtained early in the acute phase of the illness [19,20]. Overall, the positive rate of virus isolation for enteroviruses from throat swabs in acute period is less than 50%. Even combined throat plus vesicle swabs enables the identification of virus increase, but still less than 70% [9,13]. It is obvious that measurement of EV71 shedding merely based on virus culture will result in a portion of false negative. Certainly, identification of positive EV71 nucleotides by PCR in specimen does not indicate the presence of 100% live virus. Therefore, combination of the results from those two techniques may be more helpful for evaluating EV71 shedding. Additionally, detection of virus in a sample does not equal to being able to set up an efficient infection or transmission, which may be influenced by numerous factors, e.g virus load, exposing time and pathway, environmental and host situations.

In line with the concept that the EV shedding time from gastrointestinal tract usually longer than from respiratory tract [13], our study also illustrates a similar pattern that more than 20% cases maintain EV71 positive in the stool samples after clearance of EV71 nucleotides in their throats. It highlights a special requirement of decontamination for feces after recovery.

## Conclusions

Excretion of EV71 may persist for months after infection, but most cases become negative 2 weeks after onset. Thus, the patients during the first 2 weeks should be at high risk to spread the pathogens.

## Competing interests

We declare that we have no financial and personal relationships with other people or organizations that can inappropriately influence our works; there is no professional or other personal interest of any nature or kind in any product, service and/or company that could be construed as influencing the position presented in, or the review of, the manuscript entitled.

## Authors' contributions

JH and XJM collected and analyzed data. JFW participated in epidemiology investigation and blood sampling. YHL, YLH, CC, CT, CG and MW carried out PCR tests. XPD designed and coordinated the study, analyzed data and drafted the manuscript. All authors read and approved the final manuscript.

## Pre-publication history

The pre-publication history for this paper can be accessed here:

http://www.biomedcentral.com/1471-2334/10/178/prepub
